# Acidic mammalian chitinase gene is highly expressed in the special oxyntic glands of *Manis javanica*

**DOI:** 10.1002/2211-5463.12461

**Published:** 2018-07-04

**Authors:** Jing‐E Ma, Lin‐Miao Li, Hai‐Ying Jiang, Xiu‐Juan Zhang, Juan Li, Guan‐Yu Li, Jin‐Ping Chen

**Affiliations:** ^1^ Guangdong Key Laboratory of Animal Conservation and Resource Utilization Guangdong Public Laboratory of Wild Animal Conservation and Utilization Guangdong Institute of Applied Biological Resources Guangzhou China

**Keywords:** adaptation, chitin digestion, conservation, myrmecophagy, stomach

## Abstract

The Malayan pangolin (*Manis javanica*) is a mammal that feeds primarily on ants and termites, which contain the energy‐rich carbohydrate chitin. Chitin is digestible by endogenous enzymes of the typical mammalian gastrointestinal tract, especially the acidic mammalian chitinase (AMCase). The objective of this research was to determine whether AMCase activity is expressed in the stomach of *M. javanica*. The stomach tissues were divided into three parts: the gastric sack, the oxyntic glands, and the pyloric musculature, which were assayed by conventional RT‐PCR, quantitative reverse transcriptase‐coupled PCR (qPCR) and western blot. Information regarding 3D structural models of AMCase was also obtained. In conclusion, acidic mammalian chitinase is highly expressed in the oxyntic glands of the *M. javanica* species.

**Abbreviations**
AMC: aseacidic mammalian chitinase
CITES I: Convention on International Trade in the Endangered Species of Wild Fauna and Flora
CT: threshold cycle
GI: gastrointestinal
IUCN: International Union for Conservation of Nature
nsSNPs: nonsynonymous single nucleotide polymorphisms
qPCR: quantitative reverse transcriptase‐coupled PCR

The Malayan pangolin (*Manis javanica*) is an unusual mammal, as it is a scale‐covered, toothless specialist myrmecophage that feeds mostly on ants and termites. It is found mainly in South‐East Asia 1. *M. javanica* is classified as critically endangered by the International Union for Conservation of Nature (IUCN) Red List of Threatened Species^®^ and has been included in appendix I of the Convention on International Trade in the Endangered Species of Wild Fauna and Flora (CITES I) reference 2. Pangolins feed primarily on ants and termites 3, 4, 5, 6, 7. Both ants and termites are covered by chitin teguments. In most cases, the hard cover polysaccharide chitin of insects accounts for 5–20% of their dry weight 8 and may be an important component of the pangolin daily diet. However, the health of pangolins improves when additional chitin is included in their diet 9, similar to findings observed in giant anteaters (*Myrmecophaga tridactyla*) 10, 11, thus suggesting that their gastrointestinal (GI) tract is highly adapted to chitin digestion. This observation also led us to ask whether *M. javanica* can actually digest chitin or whether it simply serves as dietetic fiber.

Chitin can be degraded by chitinases and catabolized into digestible components, such as *N*‐acetyl‐glucosamine. In mammals, only two active chitinases have been identified 12, 13, chitotriosidase and acidic mammalian chitinase (AMCase) 14, 15, 16. Chitotriosidase is mainly secreted by phagocytes and acts against chitin‐containing pathogens 17. AMCase is another type of active chitinase that is common and conserved among mammals. This enzyme was discovered in mice 14 and has been extensively studied in mice and humans. The pathophysiological importance of this enzyme is already published 17, 18, 19. Some studies have shown that this gene is highly expressed in gastric glands, indicating its digestive function 13, 14, 20, 21, 22, 23, 24, 25. Human body produces AMCase in the gastric epithelium, where it may function as a digestive enzyme that breaks down chitin‐containing foods, including arthropod insects, whose cuticles are composed of chitin 26. Paoletti *et al*. suggested that the higher AMCase activity in tropical human populations with higher rates of entomophagy might represent an adaptive response to alimentary habits; however, it was a hypothesis that remains to be tested 26. AMCase has also been found in the stomachs of macaques (*Macaca fascicularis*), mice (*Mus musculus*), cattle (*Bos taurus*), and rats (*Rattus norvegicus*) at the protein and RNA expression levels 14, 21, 22, 27, 28, 29. The immunohistochemical results from Strobel's study also show AMCase localization in the stomach of bat species, particularly in the stomach's chief cells and gastric chief cells, serous‐type secretory cells that gather at the bottom of gastric glands 30.

With reporting of the genomic sequences and transcriptome of *M. javanica* 31, 32, the sequence of AMCase in *M. javanica* is deposited in NCBI database (GenBank Accession No: XM_017651285.1). We suggest that AMCase is mainly expressed in the stomach of *M. javanica* and might be involved in chitin digestion. To our knowledge, some special oxyntic glands in the stomach of *M. javanica* are organized into a gland mass, forming an oval mound elevated to the gastric lumen in the middle of the greater curvature 33. Information concerning 3D structural models of the AMCase in *M. javanica* was also provided in this manuscript. Our research aimed to determine whether AMCase is expressed in the stomach of *M. javanica*, especially in the oxyntic glands, and to assess whether *M. javanica* species can digest the cover of chitinous arthropods that might be related to unusual ant and termite diet of this specialist myrmecophagist.

## Materials and methods

### Ethics statement

Three female adult *M. javanica* samples were provided by the Dongguan Institute of Qingfengyuan Animal Medicine (Dongguan, Guangdong, China). The mouse stomach was provided from another study. The specimens were dissected after their natural death and stored in −80 °C until protein extraction. All animal procedures were approved by the ethics committee for animal experiments at the Guangdong Institute of Applied Biological Resources (Reference Number: G2ABR20170523) and followed basic principles.

### Phylogenetic analysis of the AMCase in *M. javanica* (PanChi)

To classify the AMCase from *M. javanica* among vertebrate chitinases, we compared the amino acid sequence from *M. javanica* (GenBank Accession No: XP_017506774.1) to the same protein in humans (GenBank Accession No: AAG60019.1), crab‐eating macaques (GenBank Accession No: ACV74253.1), house mice (GenBank Accession No: NP_075675.2), Norway rats (GenBank Accession No: NP_997469.1), golden hamsters (GenBank Accession No: XP_005076641.1), and cattle (GenBank Accession No: NP_777124.1) via sequence alignments generated using the clustalw2 program ( http://www.ebi.ac.uk/Tools/msa/clustalw2/).

### Prediction of the 3D structural models of AMCase in *M. javanica* (PanChi)

The 3D structural models of *PanChi* were predicted by the swiss‐model program ( http://swissmodel.expasy.org/), based on the template of the acidic mammalian chitinase catalytic domain of human (PDB ID: http://www.rcsb.org/pdb/search/structidSearch.do?structureId=3fy1), to obtain the structure of the catalytic domain.

### Biological samples

The gastric sack, oxyntic glands, and pyloric musculature were collected immediately after dissection, frozen in liquid nitrogen, and stored at −80 °C until RNA extraction.

### RNA isolation and RT‐PCR

Total RNA from the tissues of the three samples were extracted with the RNAiso reagent (Takara Bio, Kusatsu, Japan) and treated with DNase I (Takara Bio). The quality and quantity of RNA were assessed using denatured gel electrophoresis and NanoDrop 2000c spectrophotometry (Thermo, Waltham, MA, USA). The cDNA synthesis of mRNA was performed using a PrimeScript RT Reagent Kit (Perfect Real Time; Takara Bio).

### Quantitative reverse transcriptase‐coupled PCR (qPCR) validation

qPCR was performed at 95 °C for 10 min, followed by 40 cycles of 15 s at 95 °C, 30 s at 60 °C, and 30 s at 72 °C in a QuantStudio™ 5 Real‐Time PCR System (Life Technologies, Carlsbad, CA, USA). The PowerUp™ SYBR™ Green Master Mix (Life Technologies) was used in this assay, and β*‐actin* was amplified as an endogenous control. The primer pairs used for qPCR (Table 1) were designed by the Sangon Company (Shanghai, China), and the PCR products were visualized on 1% agarose gels and subjected to Sanger sequencing. The results of quantification are expressed as the threshold cycle (*C*_T_) value determined based on a manually adjusted baseline. The relative gene expression levels in various samples were calculated as previously described 34. Differences between the *C*_T_ values of target genes and endogenous controls were computed as ▵*C*_T_ **=* C*_T_ target − *C*_T_ endogenous control. The expression levels of the target genes relative to those of the endogenous control were determined as $2^{-\Delta C_{T}}$ . PCR was repeated three times per sample, and the average values of $2^{-\Delta C_{T}}$ were used to determine the differences in the expression levels of the target genes. All data are expressed as the means ± SEs of the mean.

**Table 1:** Primers for qPCR

mRNA target		Primer sequence (5′→3′)	Accession no. in GenBank	Product size (bp)
CHIA	F	AACATTGACCCCTGCCTCTG	XM_017651285.1	128
	R	GGCGGTTCTCAGAAGTGGAA		
β‐actin	F	CTCTACGCCAACACAGTG	XM_017664185.1	211
	R	CATACTCCTGCTTGCTGAT		

### Immunoblotting

Tissue proteins from the three samples were prepared as follows: (a) The tissues were dissected on ice, and the excess blood was wash away; (b) the fresh tissue was placed in a clean mortar and ground in liquid nitrogen; (c) the tissue powder was ground into a precooling tube; (d) an appropriate cell lysis buffer was added, containing 20 mm Tris (pH 7.5), 150 mm NaCl, 1% Triton X‐100, and several protein inhibitors such as sodium pyrophosphate, β‐glycerophosphate, EDTA, Na_3_VO_4_, and leupeptin (Beyotime Biotechnology, Shanghai, China) according to the amount of tissue (1 mL for 0.1 mg tissue); (e) the supernatant was sonicated on ice for 15–20 min; (f) spin at 12 000 rpm for 15–20 min at 4 °C; and (g) the supernatant was transferred into a new tube that was kept on ice at all times. The protein content in the supernatant was quantified using a Bradford protein assay kit (Beyotime Biotechnology). Proteins were separated by electrophoresis on 10% SDS/polyacrylamide gels, transferred to nitrocellulose membranes (Beyotime Biotechnology) at 100 V for 45 min, and blotted with a polyclonal AMCase antibody (1:1000, CSB‐PA874858ESR2HU, Wuhan Huamei Biology, WuHan, China, http://www.cusabio.cn/product/Polyclonal_Antibody/CHIA_Antibody-1165081.html) and a polyclonal β‐actin antibody (1:1000, CSB‐PA001205LA01HU, Wuhan Huamei Biology, http://www.cusabio.cn/product/Polyclonal_Antibody/ACTA1_Antibody-1027890.html) at room temperature for 1 h. The membranes were rinsed three times with TBST and incubated with goat anti‐rabbit IgG (Jackson, West Grove, PA, USA) diluted 1:2000 at room temperature for 45 min. After three washes with TBST, the membranes were developed using the BeyoECL Star kit (Beyotime Biotechnology). The blots were washed four times, and antibody binding was visualized using odyssey fc ® imager (LI‐COR Biosciences, Lincoln, NE, USA) and image studio western analysis software.

## Results

### Macroscopic observation

**Figure 1:**
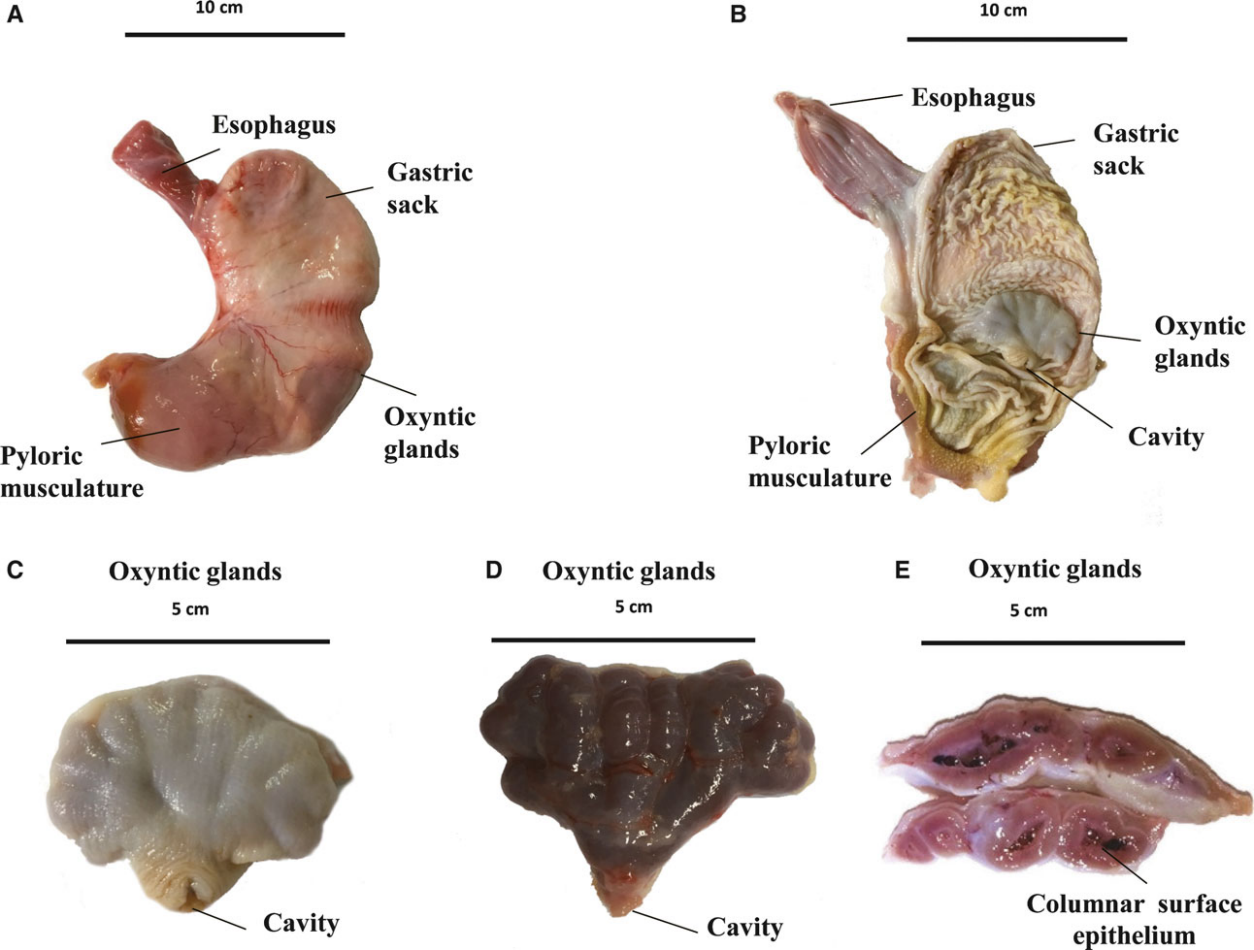
Organ arrangement and stomach appearance of *M. javanica*. (A) Arrangement of stomach organs and their appearance. (B) Transverse sections of stomach organs. (C) Appearance of the oxyntic gland tissue. (D) Appearance of oxyntic gland tissue after being peeled. (E) Transverse sections of oxyntic gland tissue.

The shape of the pangolin stomach is similar to that of a pea pod and can be divided into three sections: the gastric sack, oxyntic gland, and pyloric musculature (Fig. 1A). The stomach wall of the gastric sack is relatively loose and contains many folds (Fig. 1B). The oxyntic glands are located at the bottom of the stomach and consist of a simple columnar surface epithelium (Fig. 1B–E). The oxyntic glands open into a common cavity, which faces the pylorus. The pyloric muscle region is an inflated muscular tissue with yellow horny epithelial tissue, which is similar to the stomach muscle of birds (Fig. 1B).

### Molecular analysis of the AMCase

Sequence alignment analysis revealed that the deduced AMCase amino acid sequences of *M. javanica* (PanChi) showed high identity (79.7%–88.0%) to those of other mammals, such as humans, crab‐eating macaques, house mice, Norway rats, golden hamsters, and cattle. PanChi showed 88.0% identity to the AMCase of humans.

**Figure 2:**
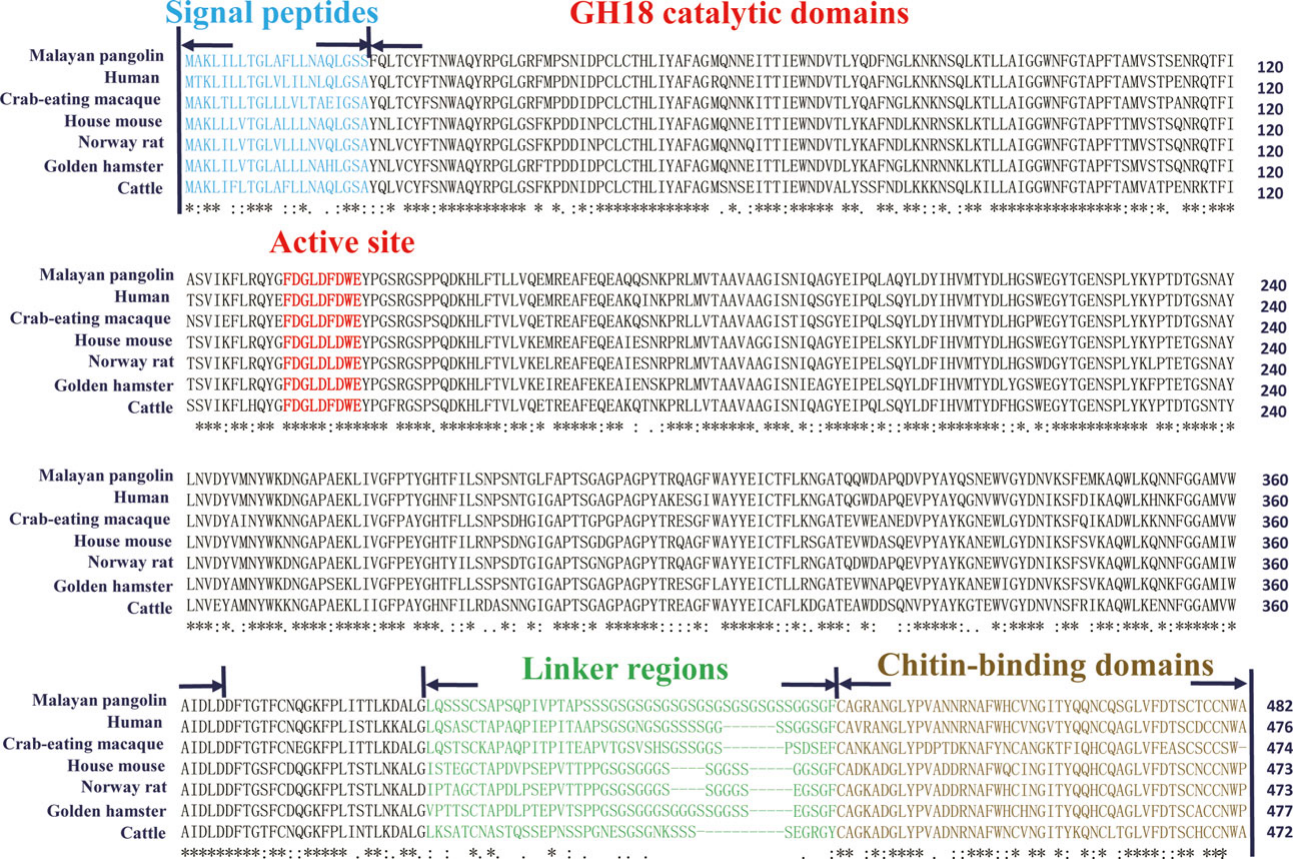
Amino acid sequence alignment of AMCase from *M. javanica* (PanChi), with those of other mammal, including humans, crab‐eating macaques, house mice, Norway rats, golden hamsters, and cattle. GenBank Accession Nos.: http://www.ncbi.nlm.nih.gov/protein/AAG60019.1, http://www.ncbi.nlm.nih.gov/protein/ACV74253.1, http://www.ncbi.nlm.nih.gov/protein/NP_075675.2, http://www.ncbi.nlm.nih.gov/protein/NP_997469.1, XP_005076641.1, and http://www.ncbi.nlm.nih.gov/protein/NP_777124.1. Identical residues of N‐terminal signal peptides are shown with blue letters, the active sites are shown with red letters, the linker regions are shown with green letters, and C‐terminal chitin‐binding domains are shown with yellow letters.

PanChi contained N‐terminal signal peptides, GH18 catalytic domains, linker regions, and C‐terminal chitin‐binding domains (Fig. 2). The linker regions of the PanChi were longest among the AMCases identified in the six species, which might indicate that a better ability to digest chitin. Parts of the GH18 catalytic domain amino acid sequences of both enzymes were detected as the active site motif sequence of ‘DXXDXDXE’, which conserves the sequences of the 18 GH family chitinases, including other AMCases in the six species (Fig. 2).

### Prediction of the 3D structural models of PanChi

**Figure 3:**
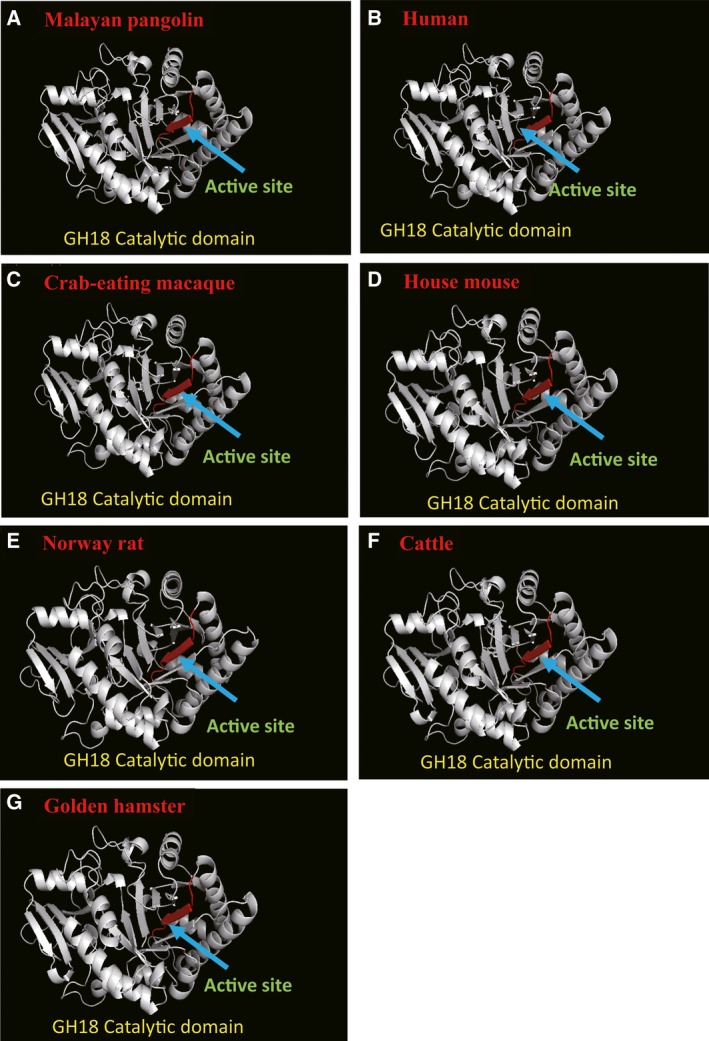
3D structure prediction models of AMCase in seven species, including Malayan pangolins (A), humans (B), crab‐eating macaques (C), Norway rats (D), house mice (E), cattle (F), and golden hamsters (G). 3D structural models were predicted using SWISS‐MODEL ( http://swissmodel.expasy.org/). The structure of the catalytic domain of acidic mammalian chitinase from Homo sapiens (Protein Data Bank [PDB] ID: http://www.rcsb.org/pdb/search/structidSearch.do?structureId=3FY1) was used as the template.

We predicted the 3D structures of PanChi using the structure of the catalytic domain of the acidic mammalian chitinase from Homo sapiens (Protein Data Bank [PDB] ID: http://www.rcsb.org/pdb/search/structidSearch.do?structureId=3FY1). PanChi has catalytic domains that are similar to those of other analogs in structure. This structure is thought to be characteristic of GH18 family chitinases. The active site is marked in Fig. 3. These results suggested that PanChi efficiently degraded crystalline chitin and exhibited particularly high activity toward chitin nanofibers.

PanChi has the longest linker regions among the acid mammal chitinases identified to date (Fig. 2). PanChi was absorbed by affinity columns when chitin was used as a carrier. Our findings herein also suggested that the catalytic domain of PanChi can hydrolyze a wide variety of chitins using the longest linker region and the chitin‐binding domain. This mechanism may underlie its high chitinase activity toward crystalline chitins.

#### mRNA expression level of AMCase in digestive tissues

**Figure 4:**
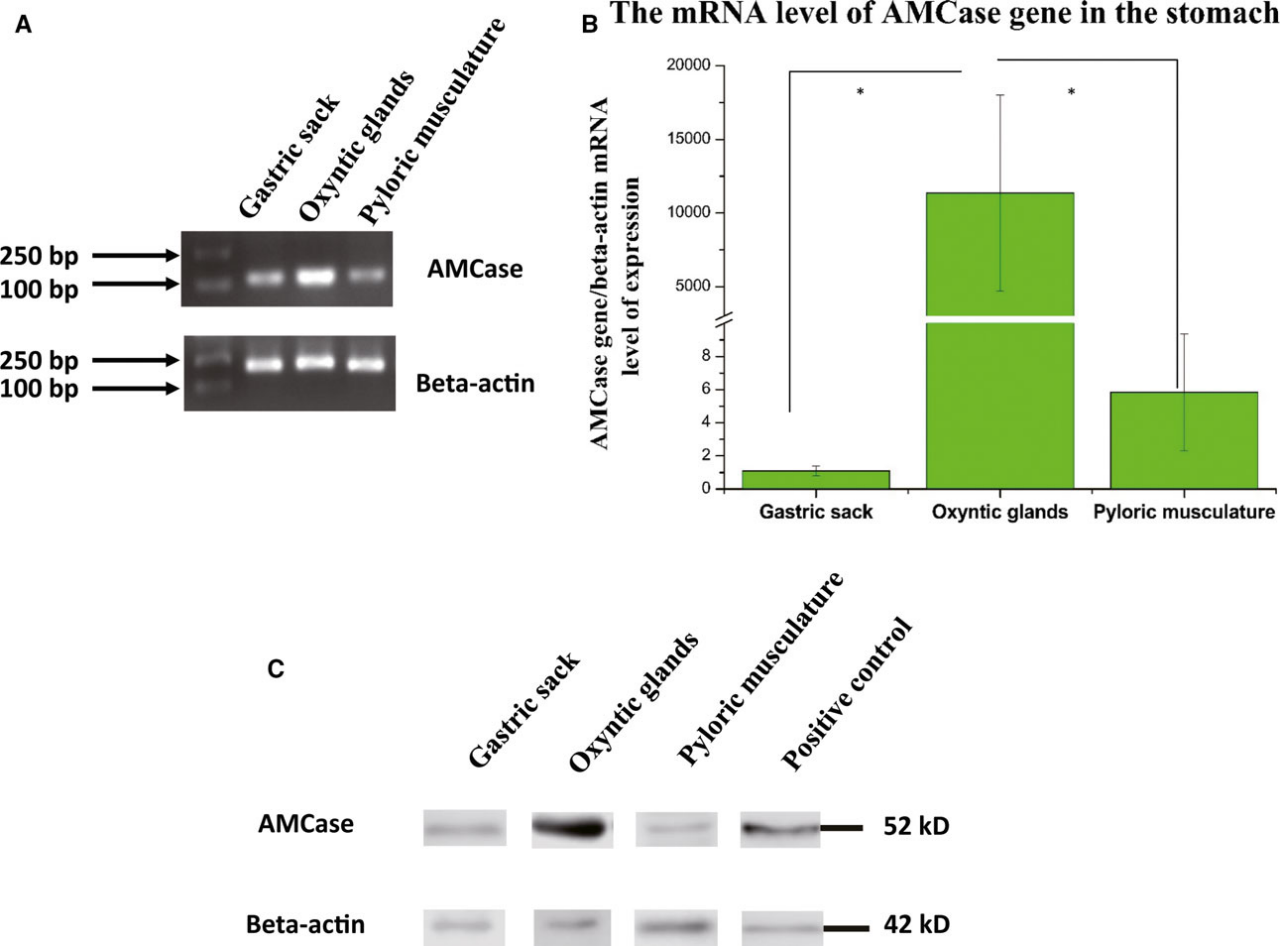
(A) mRNA expression of AMCase and β‐actin in the gastric sack, oxyntic glands, and pyloric musculature as determined by conventional RT‐PCR. (B) Relative mRNA levels of AMCase in the gastric sack, oxyntic glands, and pyloric musculature from three adult *M. javanica* as determined with qPCR. Data are presented as the means ± SDs (*n* = 3). Asterisks indicate a significant difference between the two indicated groups, and * indicates *P* < 0.05. (C) Expression of AMCase in the oxyntic glands of *M. javanica*. Relative protein levels of AMCase in oxyntic glands from *M. javanica* and *Mus musculus* stomach were determined by western blot analysis.

To study the expression levels of *AMCase* in *M. javanica in vivo*, total RNA from the stomach tissues was analyzed using qPCR assays, and β*‐actin* was selected as the endogenous control. *AMCase* mRNAs were expressed in the stomach tissues, mainly in oxyntic glands (Fig. 4A,B).

#### Protein expression levels of AMCase in digestive tissues

Western blot analysis of the oxyntic glands showed a characteristic band at a molecular weight of 52 k, consistent with the presence of AMCase (Fig. 4C). This protein band was also detected in the *Mus musculus* stomach, which was positive for anti‐AMCase 30. The expression level of AMCase was also compared to that of the housekeeping gene‐β‐actin (Fig. 4D).

## Discussion

Compared with the amino acid sequences of AMCase that have been identified in humans, crab‐eating macaques, house mice, Norway rats, golden hamsters, and cattle, that of AMCase in *M. javanica* showed a high level of homology. The high conservation of AMCase throughout evolution suggests these proteins have important functions. Its three‐dimensional structure suggested that AMCase is an active enzyme.

AMCase mRNA was expressed mainly in the oxyntic glands, and the AMCase protein was also present in the oxyntic glands. This was not unexpected because *M. javanica* eats a significant amount of chitin‐containing foods. These results suggested that AMCase secreted from oxyntic glands may digest chitin into fragments within the stomach. Herein, we first determined that AMCase expression was localized in the stomach. The oxyntic glands at the bottom of the stomach showed high expression of AMCase in *M. javanica*.

As previously shown for the stomach AMCase of *M. musculus* 14, 22, 27, chief cells secrete digestive enzymes located in numerous cytoplasmic granules 30. Goto *et al*. demonstrated that the production site of stomach AMCase in *M. musculus* was the secretory granules 28, this was consistent with the finding that these AMCase enzymes were mainly secreted by the gastric chief cells in bat species 30. Strobel's study also clearly demonstrated that European insectivorous bats of the Vespertilionidae family have the digestive enzyme AMCase, which was active and located in the stomach, particularly in or around chief cells at the base of the gastric glands 30. Human AMCase activity was present in gastric juices of 20 Italian patients, and the absence of activity in 20% of the gastric juices might be a consequence of virtual absence of chitinous food in the Western diet. This suggested that the high AMCase activity in tropical human populations with higher rates of entomophagy might represent an adaptive response to alimentary habits, which still needed to be proven 26. Furthermore, AMCase enzymatic activity might be genetically regulated in human by nonsynonymous single nucleotide polymorphisms (nsSNPs), which might be critical for both pH specificity and substrate binding 35, 36. Insect‐eating primates share an adaptation found in insectivorous bats (*Vespertilionidae*) and mice (*Mus musculus*) 13, 30, which might use the acidic mammalian chitinase to digest chitin in insect exoskeletons. The efficient digestion of insect exoskeletons likely has important adaptive benefits for all insect‐eating primates, mice, and insectivorous bats 20. However, the results of our study indicate high AMCase expression in special oxyntic glands of the *M. javanica* stomach.

In conclusion, *M. javanica* has an acidic mammalian chitinase that is produced mainly in the oxyntic glands of the stomach. However, high expression of AMCase was observed in special oxyntic glands of the *M. javanica* stomach, which was mostly consistent with previous studies 13, 20, 30. These results suggested that the gastrointestinal tracts of *M. javanica* had evolved an enzymatic adaptation allowing specialized myrmecophagy. However, further examination is needed to clarify the mechanism of AMCase functions in *M. javanica*. Further studies are necessary to reveal chitinolytic bacteria in the intestinal, which might also play an important role in nutrient intake and food digestion. The results in the study provide a theoretical basis for why chitin is an integral part of the pangolin diet and may provide a basis for the protection of this endangered species.

## Author contributions

J‐EM designed the assays and wrote the drafted manuscript; L‐ML, H‐YJ, and X‐JZ prepared the materials; JL and G‐YL analyzed data; J‐PC revised the drafted manuscript.
